# Association between unemployment rates and prescription drug utilization in the United States, 2007–2010

**DOI:** 10.1186/1472-6963-12-435

**Published:** 2012-11-30

**Authors:** Daniel Kozman, Christopher Graziul, Robert Gibbons, G Caleb Alexander

**Affiliations:** 1 Pritzker School of Medicine, University of Chicago, Chicago, IL, USA; 2 Department of Sociology, University of Chicago, Chicago, IL, USA; 3 Department of Hospital Medicine, University of Chicago, Chicago, IL, USA; 4 Department of Health Studies, University of Chicago, Chicago, IL, USA; 5 Center for Health Statistics, University of Chicago, Chicago, IL, USA; 6 Department of Epidemiology, Johns Hopkins Bloomberg School of Public Health, Baltimore, MD, 21205, USA; 7 Department of Medicine, Johns Hopkins Medicine, Baltimore, MD, USA; 8 Department of Pharmacy Practice, University of Illinois at Chicago School of Pharmacy, Chicago, IL, USA

**Keywords:** Economic recession, Pharmacoeconomics, Pharmacoepidemiology, Public health

## Abstract

**Background:**

While extensive evidence suggests that the economic recession has had far reaching effects on many economic sectors, little is known regarding its impact on prescription drug utilization. The purpose of this study is to describe the association between state-level unemployment rates and retail sales of seven therapeutic classes (statins, antidepressants, antipsychotics, angiotensin-converting enzyme [ACE] inhibitors, opiates, phosphodiesterase [PDE] inhibitors and oral contraceptives) in the United States.

**Methods:**

Using a retrospective mixed ecological design, we examined retail prescription sales using IMS Health Xponent™ from September 2007 through July 2010, and we used the Bureau of Labor Statistics to derive population-based rates and mixed-effects modeling with state-level controls to examine the association between unemployment and utilization. Our main outcome measure was state-level utilization per 100,000 people for each class.

**Results:**

Monthly unemployment levels and rates of use of each class varied substantially across the states. There were no statistically significant associations between use of ACE inhibitors or SSRIs/SNRIs and average unemployment in analyses across states, while for opioids and PDE inhibitors there were small statistically significant direct associations, and for the remaining classes inverse associations. Analyses using each state as its own control collectively exhibited statistically significant positive associations between increases in unemployment and prescription drug utilization for five of seven areas examined. This relationship was greatest for statins (on average, a 4% increase in utilization per 1% increased unemployment) and PDE inhibitors (3% increase in utilization per 1% increased unemployment), and lower for oral contraceptives and atypical antipsychotics.

**Conclusion:**

We found no evidence of an association between increasing unemployment and decreasing prescription utilization, suggesting that any effects of the recent economic recession have been mitigated by other market forces.

## Background

The recent economic recession has heightened awareness regarding the effects of unemployment on health care utilization and health outcomes. There are a variety of causal pathways and effects that are possible. For example, rising unemployment may lead to increased rates of illness, including hypertension [1], hypercholesterolemia [2], and other risk factors for cardiovascular disease [3]. Increased unemployment has also been associated with increased practice of deleterious health behaviors, such as smoking [4], heavy drinking [5], and reduced amounts of physical activity [6]. These mitigating factors may contribute to increased health care utilization. By contrast, individuals may reduce their health care spending and utilization through an “income effect” or “insurance effect” [7,8]. While COBRA does blunt the immediate impact of new unemployment on insurance status, a prolonged recession may prevent expedient reestablishment of employment, thus increasing potential gaps in coverage. Use of recommended preventive services such as colonoscopy may concurrently decline [9]. Such decreases in utilization might be particularly evident when examining prescription drug use, since prescription drugs are widely used, linked with the delivery of ambulatory care, and the out-of-pocket costs for these therapies can be considerable.

Although little is known regarding how the recent economic recession has affected prescription drug utilization, several studies have examined the effects of the recession on other components of the health care sector (Table 1). For example, the number of uninsured nonelderly Americans increased by 5.6 million between 2007 and 2009 [10], and over a quarter of Americans reported reducing their routine medical care use during this recession [11]. Over the same period, deductibles, copayments for office visits, and prescription drug copayments increased [12]. These changes also corresponded to a slowing of health care spending growth, which reached a rate in 2008 of 4.4%, the slowest rate of growth over the previous 48 years [13]. One cause of this slowing has been reduced private health insurance enrollment as a result of a continuing high unemployment and the expiration of subsidies for coverage provided through COBRA [14].

**Table 1:** Select examples of studies examining the association between the recession and health care utilization

** STUDY**	**OBJECTIVE**	**DATA AND METHODS**	**KEY FINDINGS**
Holahan J	To quantify changes in health insurance coverage seen during the recent recession	Data from the Census Bureau’s Current Population Survey (CPS) used to describe trends in coverage	· From 2007–09, number of uninsured nonelderly Americans increased by 5.6 million, from 45 to 50 million
			· Midwest experienced much larger drop in employer-sponsored insurance and smaller increases in public insurance than Northeast
Lusardi A et al.	To study the association between shocks to resources and changes in routine medical care Usage	Data from nationally representative survey of individuals 18–65 years of age in the US, UK, France, Germany, and Canada during 2009 using multivariate regression models	· Individuals whose wealth fell by 30-50% were more likely to have reduced routine medical care use relative to individuals who experienced a smaller or no loss of wealth
			· Stronger effects were found among the unemployed and in the U.S. than other countries; more than a quarter of Americans reported reducing their routine medical care use during the current economic recession
Fronstin P	To examine the extent of health insurance coverage prior to and during the recession	Data from Survey of Income and Program Participation used to describe monthly changes in coverage prior to and during the recession, with emphasis on period between Sept 2007 and April 2009	· Among workers with employee-only coverage, average deductible in large firms increased from $254 in 2005 to $478 in 2009, an 88% increase, while in small firms it increased from $469 to $1,040, a 122% increase.
			· Younger workers were more likely to lose insurance coverage than older workers. Workers ages 45–54 experienced the largest increase in the percentage uninsured, increasing from 8.6% to 13.1% (or 52.3 percent) while older workers experienced the smallest increase, 8.2%.
Hartman et al.	To examine the impact of the recession on trends in U.S. health spending	Data from annual cross-sectional analysis of U.S. health spending used to describe trends	· In 2008, U.S. health care spending growth slowed to 4.4%, the slowest rate of growth over the previous 48 years
			· Deceleration was broadly based for nearly all payers and health care goods and services, as growth in both price and non-price factors slowed
Truffer et al.	To examine the impact of the recession on trends in U.S. health spending	Data from annual cross-sectional analysis of U.S. health spending used to generate projections with actuarial and econometric modeling	· Slow down in rate of private health spending growth from 2009–2010 due to reduced private health insurance enrollment, which is a result of a continuing high rate of unemployment and the expiration of subsidies for coverage provided through COBRA

In addition to investigations examining the effects of the recent economic recession, there is a large literature examining how prescription drug utilization changes based on the prices that individuals are exposed to. Many of these studies use quasi-experimental designs to examine the association between a policy change such as a change in copayments and changes in prescription utilization. Some of these studies suggest that relatively small increases in copayments may lead to clinically significant reductions in prescription utilization, and that these decreases may occur not only for non-essential treatments but also for essential medications such as insulin, thiazides and furosemide [15]. Patients with low incomes, multiple chronic health problems or no prescription drug coverage may be particularly susceptible to such “cost-related non-adherence” [16], although there are a number of other factors that mediate these associations as well [17]. Despite the insights from these studies, they focus on a specific policy change among a selected population, rather than a general deterioration in the economic climate such as has accompanied the recent economic recession.

We examined the association between state unemployment rates and retail prescription drug use over a period of thirty-five-months. We focused on the use of seven therapeutic classes - statins, antidepressants, antipsychotics, Angiotensin-converting enzyme [ACE] inhibitors, opiates, phosphodiesterase (PDE) inhibitors, and oral contraceptives - that differ in terms of their indications, target population and importance of consistent use. We hypothesized *a priori* that increasing unemployment would be associated with increasing use of some therapies such as oral contraceptives, believing that unplanned pregnancy would be even more carefully avoided in times of limited resources as suggested by prior studies [18]. On the other hand, we also predicted a decrease in use of other therapies such as PDE inhibitors and statins that are used for “lifestyle” or asymptomatic chronic conditions, as these medications may be deemed less essential from the patient’s perspective when choosing between these therapies and other important everyday commodities [19]. Meanwhile, the use of psychotropic medications was predicted to increase with unemployment, as economic hardship has previously been associated with poor mental health [20]. In addition to examining the association between unemployment rates and medication use, our data also allowed for us to examine variation in rates of prescription utilization across states and over time.

## Methods

### Study design and state unemployment data

We used a mixed ecological design for our retrospective analysis, focusing on the association between local economic recession and outcome measures of prescription drug utilization both across states and within states over time. We collected state-level monthly unemployment rates from 2007 to 2010 from the U.S. Bureau of Labor Statistics [21]. This data served as a primary indicator of the degree to which a specific state had experienced economic hardship due to the recession.

### Data on prescription drug utilization

We used the IMS Health Xponent^TM^ database in order to derive information about prescription drug sales. These data, which have been previously described [22,23], provide a highly representative sample of nationwide pharmaceutical dispensing in the United States, using records from over 38,000 of the estimated 57,000 retail stores (more than a 70% sample), 119 of the 327 existing mail service pharmacy outlets, and 820 of the estimated 3,000 long-term care pharmacies. Xponent^TM^ includes both new and refilled prescriptions issued daily from each of these dispensaries, with data aggregated for stratification by geographic region, patient age, patient gender, patient copayment, and method of payment, including cash payment.

From this data we acquired state-level monthly estimates of retail dispensed prescription drug utilization across the therapeutic classes of interest from September 2007 to July 2010. Our inclusion of seven therapeutic areas allowed us to examine the primary association of interest for drugs that differed along a number of important dimensions, including: (1) use on an as-needed vs. standing basis, (2) use for psychiatric vs. non-psychiatric illness, (3) use for symptomatic vs. non-symptomatic conditions, and (4) use for chronic disease vs. use for conditions generally treated with therapies regarded as “life-style medications”. Our retail dispensed prescription data also included sociodemographic variables that allowed for stratified analyses with respect to patient sex and age. We merged these data with publicly available population estimates provided by the U.S. Bureau of the Census and derived from the Current Population Survey to calculate state-level rates of prescription drug utilization.

### Analysis

We first used the IMS Health Xponent^TM^ data and population estimates from the Census Bureau to examine variation in the rates of prescription use per 100,000 individuals. Next, following Gibbons et al., we used mixed-effects Poisson regression [24] and state-level control variables to decompose the overall unemployment rate effects into between-state (state mean unemployment rate) and within-state (monthly deviation from the state mean unemployment rate) effects [25] while conditioning on common factors affecting prescribing behavior. We treated the within-state effect as a random effect in the model to permit state-level variation in the associations between relative changes in unemployment rates and state-level prescription rates. This modeling approach not only allows us to differentiate states which already had higher levels of unemployment prior to the recession (i.e. Michigan) from states who had lower levels, but also allows us to estimate a relationship between average state levels of unemployment and drug utilization, capturing how this absolute economic deprivation affects drug utilization. Our within-state estimates then gauge how variation in relative economic deprivation within a state, associated with the recession, relates to drug utilization. Whereas our methods remove annual and quarterly temporal patterns in drug utilization, this approach does not remove potential confounding due to other well-characterized temporal patterns in both drug utilization and unemployment (e.g., calendar effects, autoregressive “memory” across quarters, or oscillations).

We derived empirical Bayes estimates of the state-specific effects [24]. The estimated coefficients for our mixed-effects Poisson regression models are reported in Table 2 as incidence rate ratios (IRRs). Otherwise known as rate multipliers, IRRs indicate the relative impact of a covariate on a reference incidence rate, here represented by the estimated intercept (which reports the incidence rate of drug utilization per 100,000 individuals). An IRR of exactly 1.0 indicates that change in a covariate has no impact, while an IRR of 0.75 indicates that a one unit increase of the covariate is associated with a 25% lower (0.75 times lower) rate of use compared to the base incidence rate, and an IRR of 1.25 would indicate a 25% higher (1.25 times higher) rate of use compared to the base incidence rate. Models adjusted for covariates are depicted in Additional file 1: Appendix Table S2.

**Table 2:** Estimated association between state-level unemployment and retail dispensed prescription drug utilization for seven therapeutic areas

	**Opiates**	**ACE Inhibitors**	**Statins**	**Oral contraceptives**	**Atypical Antipsychotics**	**SSRIs and SNRIs**	**PDE inhibitors**
Fixed Effects							
Base Utilization Rate (per 100,000)	5162 (4788, 5566)	3992 (3871, 4117)	4356 (4246, 4470)	8261 (8160, 8363)	3326 (3187, 3471)	1382 (624, 3059)	1183 (1168, 1199)
State Mean Unemployment ^+^	1.02 (1.01, 1.03)	0.99 (0.99, 1.00)	0.98 (0.98, 0.99)	0.97 (0.97, 0.97)	0.84 (0.84, 0.84)	1.11 (0.99, 1.23)	1.01 (1.01, 1.01)
Deviation from State Mean Unemployment ^+^	1.02 (1.00, 1.03)	1.01 (0.99, 1.02)	1.04 (1.02, 1.05)	1.01 (1.00, 1.01)	1.01 (1.00, 1.02)	1.01 (0.97, 1.05)	1.03 (1.03, 1.04)
Random Effects							
Intercept	0.0752***	0.0584***	0.0590***	0.0577***	0.1483***	0.0709***	0.0918***
Intercept-UnempDev	−0.0002	0.0009*	0.0004	0.0008*	−0.0014	0.0006	−0.0016
UnempDev	0.0002***	0.0001***	0.0003***	0.0001***	0.0002***	0.0001***	0.0005***
BIC	67410	67484	66799	58081	67876	68718	40414

^+^ Values represent incident rate ratios (95% confidence intervals); base utilization rates derived using data from IMS Health Xponent™, 2007–2010 and the U.S. Bureau of Labor Statistics; models adjusted for covariates depicted in Additional file 1: Appendix Table S2.

*BIC* = Bayesian Information Criterion, a measure of model fit; UnempDev = Deviation from State Mean Unemployment; *ACE* = Angiotensin-converting enzyme; *SSRI* = selective serotonin reuptake inhibitor; *SNRI* = serotonin-norepinephrine reuptake inhibitor; *PDE* = phosphodiesterase.

Since rates of prescription drug utilization change over time based on secular and seasonal trends, all models controlled for these trends by including dummy variables for year (2008 as reference) and season (summer as reference). We included sex, age bracket, year and season as dummy control variables. Thus, typically, the base rate of utilization represents utilization per 100,000 males, who were between 20 and 64 years old, and who filled a prescription during July through September 2008. Owing to the demographic-specific nature of some drug classes, when drug utilization for a particular combination of sex and/or age bracket was particularly low (less than 1% of other combinations) that sex and/or age bracket was removed from analysis. For example, we excluded individuals less than 20 years of age in analyses of statins and ACE inhibitors, and we excluded women from the analyses of PDE inhibitors.

We performed a variety of sensitivity analyses to examine whether our findings would differ based on our analytic approach. First, we repeated our analyses after limiting our population to individuals less than 65 years of age who paid for prescriptions with cash. We reasoned that these subjects may be more likely to manifest decreased prescription utilization with rising unemployment rates. Second, we conducted a similar sensitivity analysis focusing on only those who were commercially insured and under 65 years of age. Third, we incorporated a lag interval of three months for analysis to examine whether there was an association between a state’s unemployment rate and subsequent rates of drug utilization. Finally, we repeated analyses without our covariates to determine whether a simpler model would produce substantively similar results. Comparison to models with our covariates suggested that our covariates had a statistically significant and policy relevant attenuating effect, thus we included them in the final models.

## Results

### Variation in unemployment and prescription utilization

**Figure 1:**
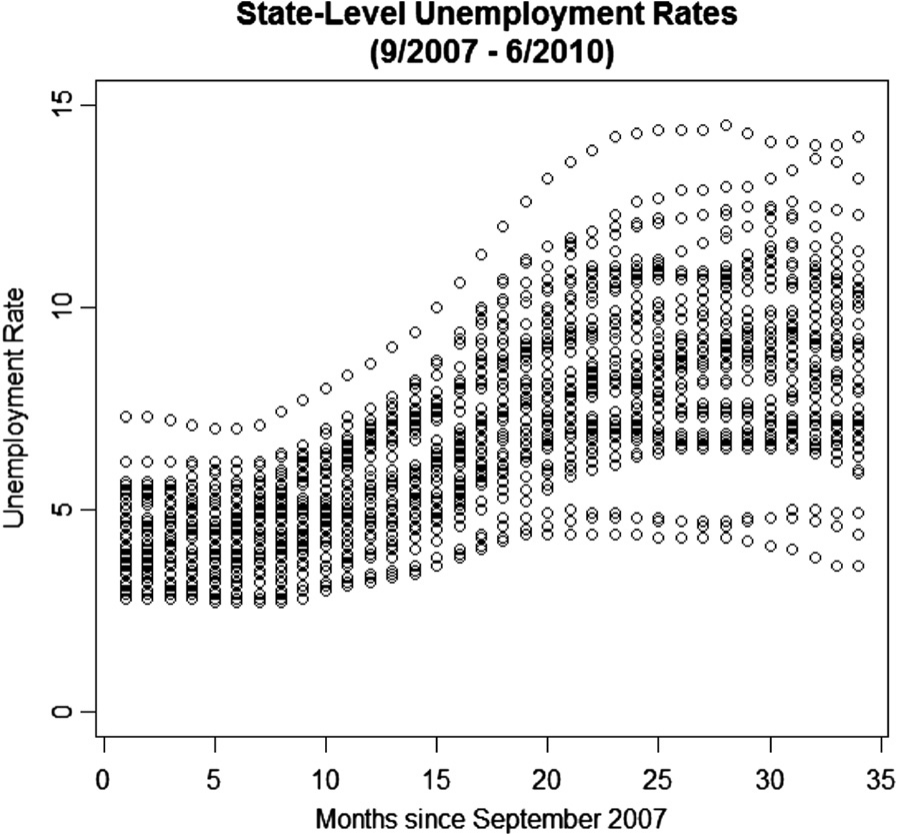
Monthly state-level unemployment rates, 2007–2010.

There was substantial variation in state level monthly unemployment during each year (Figure 1). For example, during the fourth quarter of 2007 (2007Q4), unemployment rates varied from 2.8% to 7.3%, with a mean of 4.4% and standard deviation of 1.0%. As the recession continued, the mean level of and variance in unemployment trended higher (mean 8.7% and standard deviation of 2.1% in 2010Q1).

Rates of prescription utilization also varied considerably across the therapeutic areas examined. For example, the average rate of opioid use per 100,000 people during 2008 was approximately 5109, as compared with more moderate rates of use of SSRIs/SNRIs (4115) and statins (3929), and even lower rates of use of atypical antipsychotics (966).

For most therapeutic areas, rates of use across the states also varied substantially, with rates of use two- to threefold greater in some states than others. For example, average rates of atypical antipsychotic use varied three-fold from 497 prescriptions per 100,000 residents (Colorado) to approximately 1430 prescriptions per 100,000 residents (Connecticut and Massachusetts). States that had higher utilization of one therapy generally had higher levels of utilization of other therapies as well. In 39 states, the utilization of any two drug classes exhibited a Pearson correlation of at least 0.6, with 22 states exhibiting a minimum correlation of 0.9.

Overall use of the therapies was greater among the elderly than among non-elderly, although the elderly only accounted for 25% of all use of the therapies examined. The proportion of all use that occurred among those 65 years of age or greater was highest for statins (46%) and ACE inhibitors (42%), and lower for several of the other classes examined.

### Associations between unemployment and prescription use across states

For 2 of the 7 therapeutic classes examined (ACE inhibitors and SSRIs/SNRIs), there were no statistically significant associations between the average state-level unemployment and prescription utilization. In the case of opioids and PDE inhibitors, there were small, statistically significant positive associations. For example, when the average level of unemployment for a state increased by 1% the number of opiate prescriptions per 100,000 people increased on average 2% (Table 2, “State Mean Unemployment”). Similarly, when the average level of unemployment for a state increased by 1% PDE inhibitor utilization increased on average 1%. An inverse relationship existed for statins, contraceptives and atypical antipsychotics, exhibiting decreases of 2%, 3% and 16% respectively in the rate of utilization per 1% increase in unemployment.

### Associations between unemployment and prescription use within states

Analyses of the association between prescription use and change in unemployment relative to the state mean allowed for each state to serve as its own control. Overall, states collectively exhibited statistically significant positive associations between increases in unemployment and utilization of the therapies examined (Table 2, “Deviation from State Mean Unemployment”). This association was strongest for statins (on average, a 4% increase in utilization per 1% increase in unemployment) and PDE inhibitors (on average, a 3% increase in utilization per 1% increase in unemployment), was weaker for opiates, oral contraceptives, and atypical antipsychotics, and was not statistically significant in the case of ACE inhibitors and SSRIs/SNRIs.

**Figure 2:**
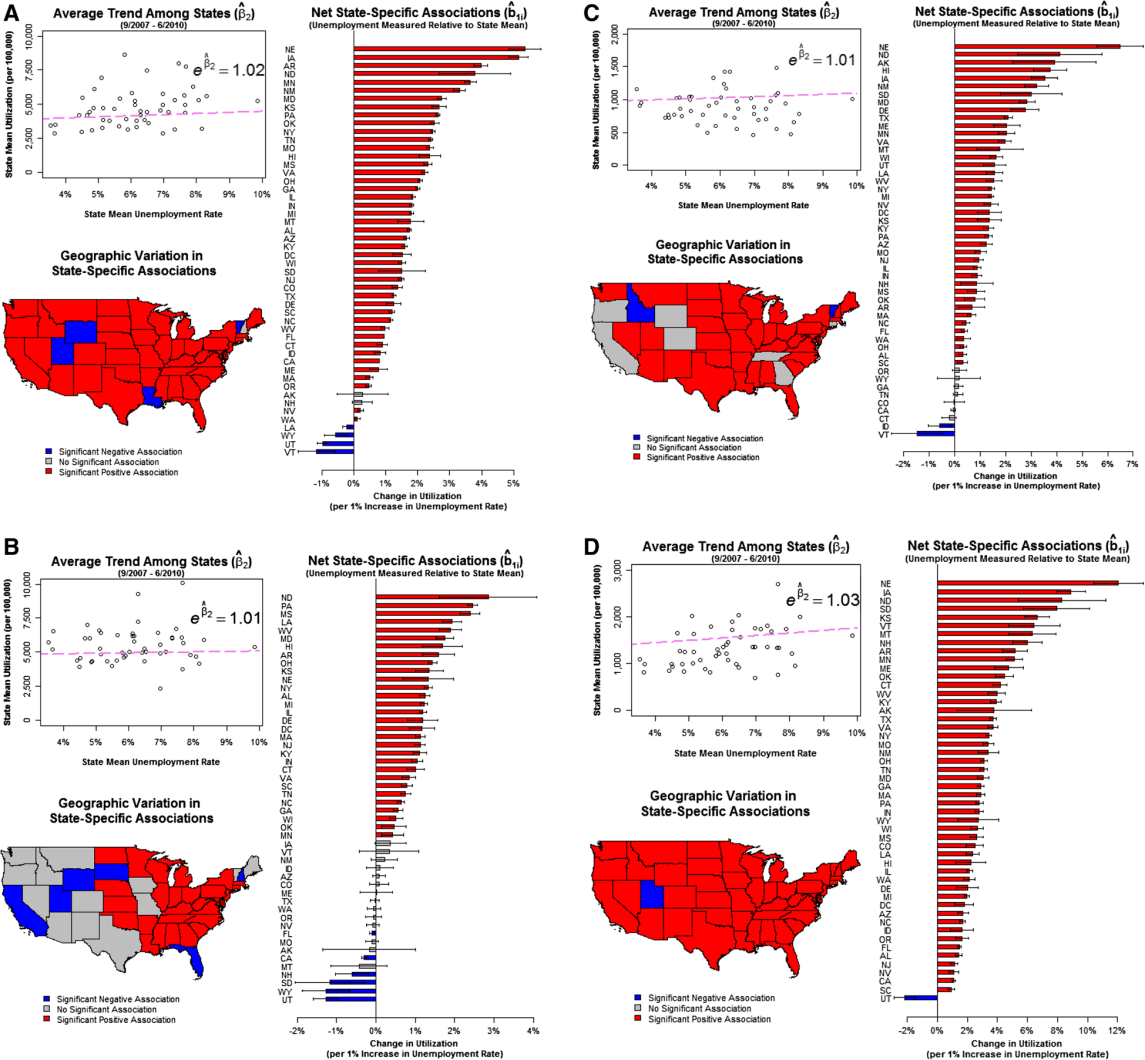
**A. Association between unemployment and state-level opioid utilization, 2007–2010. B**. Association between unemployment and state-level oral contraceptive utilization, 2007–2010. **C**. Association between unemployment and state-level atypical antipsychotic utilization, 2007–2010. **D**. association between unemployment and state-level pde inhibitor utilization, 2007–2010.

Figure 2A depicts these relationships for opiates. In 44 states, increased unemployment relative to the state mean was associated with an increase in opiate utilization. This varied from a high of approximately 5% more prescriptions per 100,000 residents (Nebraska and Ohio) to 0.1% more prescriptions (Washington) for each 1% increase in unemployment. Figures 2B, 2C, and 2D depict the associations for other therapeutic areas. For example, Figure 2B reflects the association between unemployment and oral contraceptives use, demonstrating a modest 0.6% average increase in the number of prescriptions per 100,000 residents across all states for each 1% increase in unemployment. This average net increase ranged from a 2.5% increase (North Dakota) to a 1.2% decrease (Utah). Similar trends in the net association were observed with atypical antipsychotics (Figure 2C) and PDE-inhibitors (Figure 2D), albeit with less variation in the direction of the relationship across states in the latter case.

### Sensitivity analyses

These analyses comparing the findings for the population of interest with those for the Medicare-insured population, and with those for the cash-paying and commercially insured populations under age 65, yielded no change in the trends observed in the data. The incorporation of a lag interval also did not alter our main findings or their substantive interpretation.

## Discussion

In this mixed ecological study analyzing prescription drug use for each of the 50 states from September 2007 through July 2010, we observed wide variation in aggregate utilization rates both across states and across the drug classes of interest. Differences in utilization rates across states were particularly large for statins and ACE inhibitors, where some states had as much as five-fold greater use, though each therapeutic class of interest exhibited at least two- to threefold higher rates of use in some states than others. Generally, higher utilization rates in one class were accompanied by higher utilization rates in the other classes. This across-state variation was present during every year of observation and did not change or diminish as a result of the recession. Ours is not the first study to suggest regional variation in prescription drug utilization [26-29], although in contrast to most other work we examined a variety of sources of payment as well as therapies differing along a number of important clinical dimensions. Although our mixed effects models suggested that the association between unemployment and prescription use varied remarkably across states, we found no evidence of substantial declines in prescription utilization associated with the economic recession. Given the profound and far-reaching effects of the recent economic recession in a variety of economic sectors, these findings are important since little is known about how the recession has been related to rates of drug utilization.

There are several reasons that may account for the absence of any discernible effect of the recession on rates of prescription drug utilization. First, although our analyses included terms to account for seasonal and secular trends in prescription drug utilization, we did not statistically adjust for other market forces that may have helped to attenuate any untoward effects of the recession on individuals’ access to prescription drugs. For example, the rapid growth in the availability of generic therapies, whose market share has increased from 63% in 2006 to 78% in 2010 [30], may have helped to offset decreases in use that would otherwise have been associated with the economic hardship of the recession. Increasing availability of “$4 generic drug” programs sponsored by major retailer and “big box” stores may similarly have contributed to robust pharmaceutical sales during this period [31]. Second, it is also possible that most of the newly unemployed accounted for a relatively small share of the market for prescription drugs and thus that no effect of unemployment is seen, although our findings were similar in sensitivity analyses limited to those under 65 years of age with commercial insurance or self-pay, and the elderly accounted for only approximately one-quarter of the entire market of therapies examined. Third, there are a variety of pathways whereby economic recession may affect health and health care utilization, and thus the absence of large decreases in prescription utilization may reflect in part changes such as anticipatory behavior [32] or increased psychological, social, or physical stressors that offset decreases that one might expect in prescription utilization due to an insurance or income effect.

Our study has limitations and also leaves many questions unanswered. The greatest limitation of our report is that our ecological analyses preclude an analysis of important patient, provider, and health system characteristics that may mediate or modify the associations that we describe. Our investigation is one of states and markets, and thus should not be taken to suggest that individuals have not modified their prescription drug utilization as a result of economic hardship. Second, our data consist of prescription sales rather than actual prescription utilization, and despite our analysis of seven therapeutic areas ranging from oral contraceptives to atypical antipsychotics, it is possible that patterns of utilization of other therapies differ. Finally, as our study is ecological in nature, our findings are unable to provide any information with regard to the direction of causation in the relationship between unemployment and prescription drug utilization.

## Conclusion

Extensive evidence suggests that the economic recession has had far reaching effects on many economic sectors, including effects on rates of health insurance and health care seeking behaviors in the United States. Despite this, we found no substantial association between increasing unemployment and decreasing prescription utilization, suggesting that any untoward effects of the recent economic recession have been buffered by other market forces during the same period.

## Competing interests

GCA is a consultant for IMS Health.

## Authors’ contributions

DK and GCA conceived of the study. All authors participated in the development of the analytic approach. RG supervised and CG performed the statistical analysis. DK drafted the manuscript. All authors provided substantive revisions to the manuscript and approved the final manuscript.

## Authors’ information

DK is a fourth year student at the University of Chicago Pritzker School of Medicine. CG is a doctoral candidate in Sociology at the University of Chicago. RG is a biostatistician with expertise in environmental statistics and psychometrics as well as pharmaceutical safety. GCA is a pharmacoepidemiologist and practicing general internist now at the Johns Hopkins School of Public Health and Johns Hopkins Medicine.

## Pre-publication history

The pre-publication history for this paper can be accessed here:

http://www.biomedcentral.com/1472-6963/12/435/prepub

## Supplementary Material

Additional file 1 **Appendix.** Model specification. Click here for file
