# Supplementary material for: Association between unemployment rates and prescription drug utilization in the United States, 2007–2010
Source: BMC Health Serv Res. 2012 Nov 30;12:435. doi: 10.1186/1472-6963-12-435 (PMC3541063; doi:10.1186/1472-6963-12-435)
Supplement: Additional file 1 — Appendix. Model specification. [file 1472-6963-12-435-S1.doc]

**APPENDIX. Model Specification.**

To decompose between-state and within-state effects we employed a two-level model with random intercepts and random slopes:


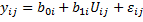


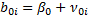


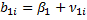


with

| 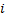 | = States, our grouping variable |
| --- | --- |
| 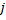 | = Month and Year (ranging from 9/2007 to 6/2010) |
| 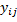 | = Utilization of Drug Class (RXs per 100,000 population) in state 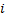 during month/year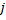 |
| 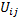 | = Unemployment rate of state 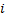 during month/year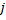 |
|  |  |

Rewriting
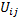
 as
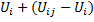
 and inserting into the original model, as outlined in Hedeker and Gibbons (2006) we arrive at


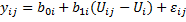


| 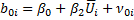  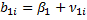 |
| --- |

with

| 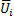 | = Mean unemployment rate of state 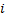 from 9/2007 to 6/2010 |
| --- | --- |
| 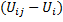 | = Deviation from mean unemployment rate of state 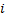 during month/year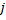 |


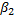
 is interpreted as the between-state effect of unemployment (or, alternatively, the effect of absolute deprivation) which represents the national trend relating unemployment to utilization.


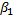
 is interpreted as the global within-state effect of unemployment (or, alternatively, the effect of relative deprivation) which represents the national trend between the relative level of unemployment (compared to a state’s mean level of unemployment) and utilization within that state.


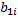
 is interpreted as the net within-state association between the deviation of the unemployment rate from the state mean (again, the effect of relative deprivation) and drug utilization within state *i*, accounting for both the global effect and any state-specific attenuations to this effect.

**APPENDIX TABLE. Estimated ASSOCIATION BETWEEN STATE-LEVEL UNEMPLOYMENT AND retail dispensed prescription drug utilization FOR SEVEN THERAPEUTIC AREAS.**

|  | Opiates | ACE Inhibitors | Statins | Oral contraceptives | Atypical Antipsychotics | SSRIs and SNRIs | PDE Inhibitors |
| --- | --- | --- | --- | --- | --- | --- | --- |
| Fixed Effects |  |  |  |  |  |  |  |
| Base utilization rate  (per 100,000 people) | 5162 (4788, 5566) | 3992 (3871, 4117) | 4356 (4246, 4470) | 8261 (8160, 8363) | 3326 (3187, 3471) | 1382 (624, 3059) | 1183 (1168, 1199) |
| Female ^+^ | 1.26 (1.26, 1.27) | 0.88 (0.88, 0.89) | 0.91 (0.90, 0.91) | --- | 1.08 (1.07, 1.10) | 2.39 (2.34, 2.45) | --- |
| Age 0-19 ^+^ | 0.13 (0.13, 0.13) | --- | --- | 0.35 (0.35, 0.35) | 0.71 (0.70, 0.73) | 0.15 (0.14, 0.16) | --- |
| Age 65 years and older ^+^ | 1.01 (1.00, 1.01) | 3.42 (3.38, 3.46) | 3.99 (3.97, 4.00) | --- | 0.81 (0.79, 0.82) | 1.08 (1.05, 1.12) | 1.35 (1.34, 1.35) |
| Spring ^+^ | 1.00 (0.99, 1.01) | 1.01 (1.00, 1.03) | 1.01 (1.00, 1.01) | 1.00 (0.99, 1.00) | 1.03 (1.01, 1.05) | 1.01 (0.98, 1.04) | 1.00 (1.00, 1.01) |
| Fall ^+^ | 0.97 (0.96, 0.98) | 0.98 (0.96, 0.99) | 0.98 (0.97, 0.99) | 0.96 (0.95, 0.96) | 0.98 (0.96, 1.01) | 0.98 (0.95, 1.02) | 0.96 (0.96, 0.97) |
| Winter ^+^ | 0.98 (0.97, 0.99) | 1.00 (0.98, 1.01) | 0.98 (0.97, 0.99) | 0.98 (0.97, 0.98) | 1.01 (0.98, 1.03) | 1.00 (0.98, 1.03) | 1.01 (1.01, 1.02) |
| 2007 ^+^ | 0.99 (0.98, 1.00) | 1.02 (1.00, 1.04) | 0.98 (0.96, 0.99) | 1.02 (1.01, 1.02) | 1.03 (1.00, 1.06) | 1.01 (0.97, 1.05) | 1.03 (1.02, 1.03) |
| 2009 ^+^ | 0.97 (0.95, 0.980 | 0.97 (0.94, 0.99) | 0.96 (0.95, 0.98) | 0.97 (0.96, 0.97) | 1.02 (0.99, 1.06) | 0.97 (0.91, 1.02) | 0.93 (0.92, 0.93) |
| 2010 ^+^ | 0.96 (0.94, 0.98) | 0.96 (0.92, 0.99) | 0.96 (0.94, 0.98) | 0.93 (0.92, 0.94) | 1.01 (0.97, 1.05) | 0.96 (0.89, 1.04) | 0.86 (0.85, 0.87) |
| State Mean Unemployment ^+^ | 1.02 (1.01, 1.03) | 0.99 (0.99, 1.00) | 0.98 (0.98, 0.99) | 0.97 (0.97, 0.97) | 0.84 (0.84, 0.84) | 1.11 (0.99, 1.23) | 1.01 (1.01, 1.01) |
| Deviation from State Mean ^+^ | 1.02 (1.00, 1.03) | 1.01 (0.99, 1.02) | 1.04 (1.02, 1.05) | 1.01 (1.00, 1.01) | 1.01 (1.00, 1.02) | 1.01 (0.97, 1.05) | 1.03 (1.03, 1.04) |
| Random Effects |  |  |  |  |  |  |  |
| Intercept | 0.0752*** | 0.0584*** | 0.0590*** | 0.0577*** | 0.1483*** | 0.0709*** | 0.0918*** |
| Intercept-UnempDev | -0.0002 | 0.0009* | 0.0004 | 0.0008* | -0.0014 | 0.0006 | -0.0016 |
| UnempDev | 0.0002*** | 0.0001*** | 0.0003*** | 0.0001*** | 0.0002*** | 0.0001*** | 0.0005*** |
| Degrees of Freedom | 10,186 | 6,786 | 6,786 | 3,387 | 10,186 | 10,186 | 3,387 |
| AIC | 67302 | 67389 | 66684 | 58001 | 67769 | 68,610 | 40334 |
| BIC | 67410 | 67484 | 66799 | 58081 | 67876 | 68718 | 40414 |

^+^ Values represent incident rate ratios (95% confidence intervals); based utilization rates derived from data from the IMS Health Xponent, 2007-2010™ and the U.S. Bureau of Labor Statistics; models adjusted for covariates depicted in Appendix Table 2

BIC=Bayesian Information Criterion, a measure of model fit; UnempDev=Deviation from State Mean Unemployment; ACE=Angiotensin-converting enzyme; SSRI=selective serotonin reuptake inhibitor; SNRI=serotonin-norepinephrine reuptake inhibitor; PDE=phosphodiesterase
